# Supplementary material for: Gesture encoding in human left precentral gyrus neuronal ensembles
Source: Commun Biol. 2025 Aug 30;8:1315. doi: 10.1038/s42003-025-08557-z (PMC12398507; doi:10.1038/s42003-025-08557-z)
Supplement: Supplementary file 2 — Supplemental Materials [file 42003_2025_8557_MOESM2_ESM.pdf]

Supplemental Materials for:
Gesture encoding in human left precentral gyrus neuronal ensembles

Carlos E. Vargas-Irwin[1,2,3], Tommy Hosman[3,4], Jacob T. Gusman [2,3,4,5], Tsam Kiu Pun[2,4,5], John D. Simeral [2,3,4], Tyler Singer-Clark[6], Anastasia Kapitonava[6], Claire Nicolas[6], Nishal P. Shah[7], Donald T. Avansino[8], Foram Kamdar[7], Ziv M. Williams[9], Jaimie M. Henderson[7,10,11], Leigh R. Hochberg[2,3,4,6]

1. Department of Neuroscience, Brown University, Providence, RI, USA
2. Robert J. and Nancy D. Carney Institute for Brain Science, Brown University, Providence, RI, USA
3. VA Center for Neurorestoration and Neurotechnology, VA Providence Healthcare System, Providence, RI, USA.
4. School of Engineering, Brown University, Providence, RI, USA
5. Biomedical Engineering Graduate Program, School of Engineering, Brown University, Providence, RI, USA.
6. Center for Neurotechnology and Neurorecovery, Department of Neurology, Massachusetts General Hospital, Harvard Medical School, Boston, MA, USA
7. Department of Neurosurgery, Stanford University, Stanford, CA, USA
8. Howard Hughes Medical Institute at Stanford University, Stanford, CA, USA
9. Department of Neurosurgery, Massachusetts General Hospital, Harvard Medical School, Boston, MA, USA.
10. Wu Tsai Neurosciences Institute, Stanford University, Stanford, CA, USA
11. Bio-X Institute, Stanford University, Stanford, CA, USA

Corresponding author email: Carlos_Vargas_Irwin@Brown.edu

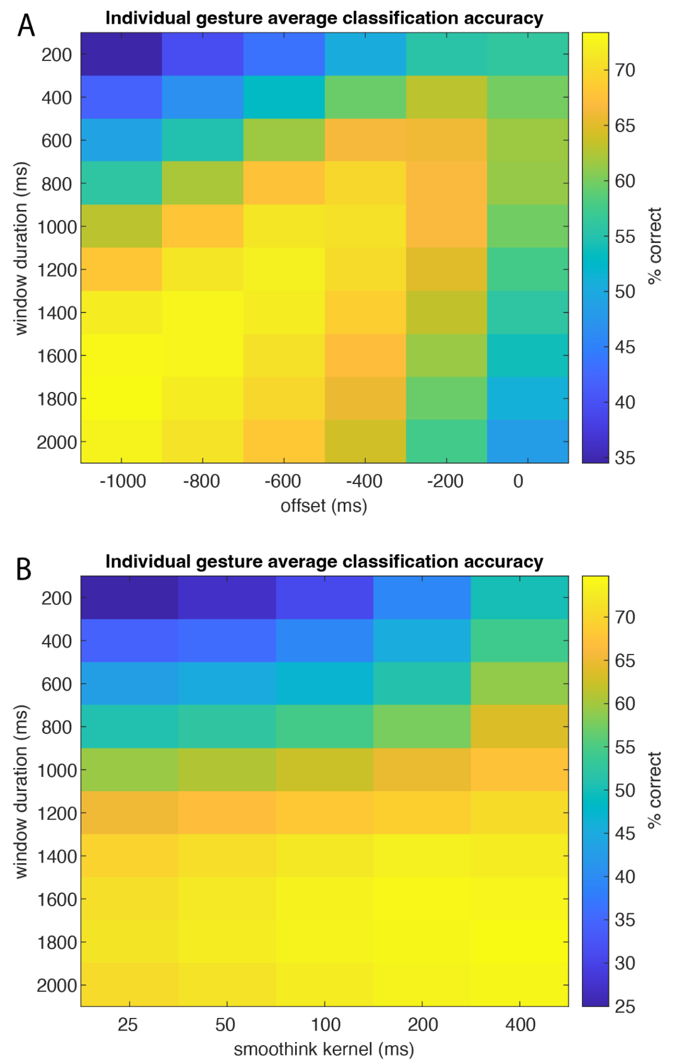

***Figure S1.* Parameter sweep for offline classification accuracy.**

We performed a parameter sweep in order to assess the effect of the time window selected for analysis and the smoothing parameters applied to the neural features. We assessed window durations between 200 and 2000ms, with offsets between 0 and -1000ms for the start of the window relative to the go cue. We also assessed the effects using gaussian kernels widths between 25 and 400ms. Overall, longer time durations and broader smoothing kernels tended to yield higher classification accuracy across the 49 gesture classes. The highest accuracy was achieved for time windows that included time periods before and after the go cue. Classification accuracy changed gradually with a broad set of parameter settings producing similar results.

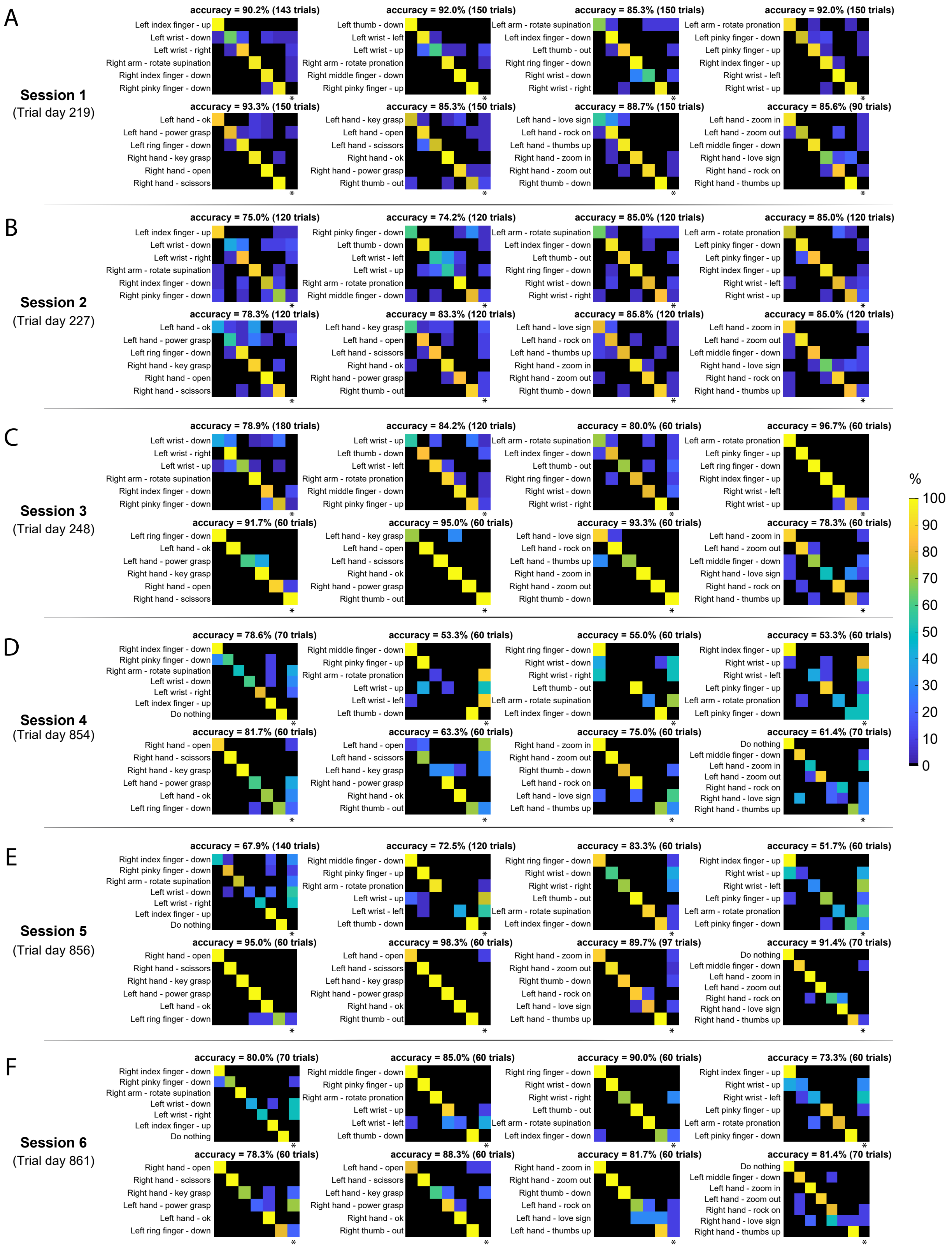

Figure S2. **Closed-loop gesture decoding for participant T11**. Each confusion matrix shows classification results between six or seven gestures in one of the eight closed-

loop gesture sets for each of the six sessions. A gesture was considered to be correctly decoded in a trial if the most frequently decoded gesture other than “no decode” matches with the cued gesture. If none of the likelihood estimations across all gestures exceeded threshold, * refers to the “no decode” class, i.e. no gesture was decoded throughout the entire trial.

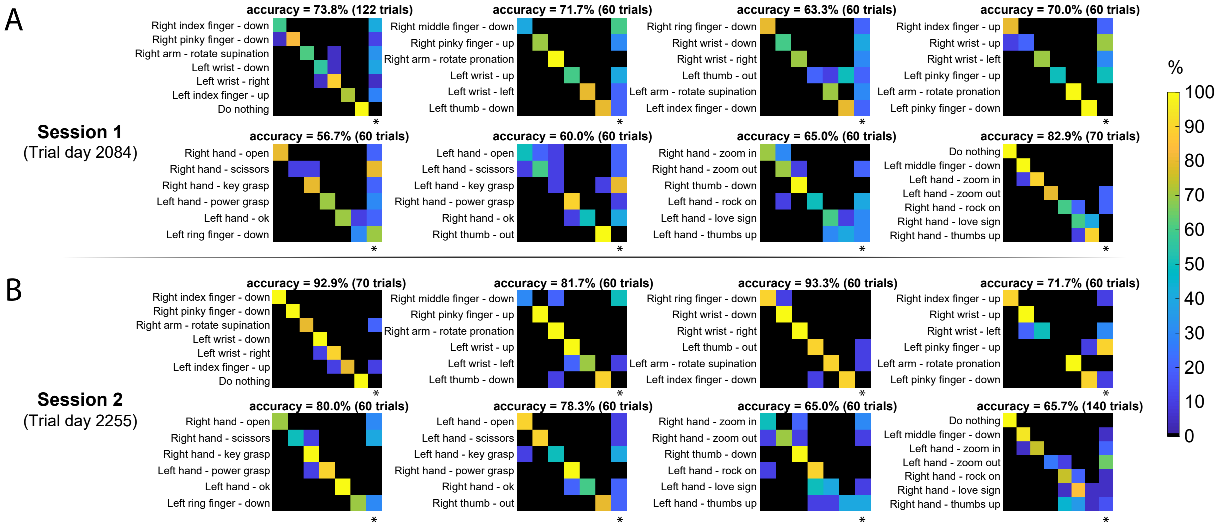

Figure S3. Closed-loop gesture decoding for participant T5. Each confusion matrix shows classification results between six or seven gestures in one of the eight closed-loop gesture sets for each of the two sessions.

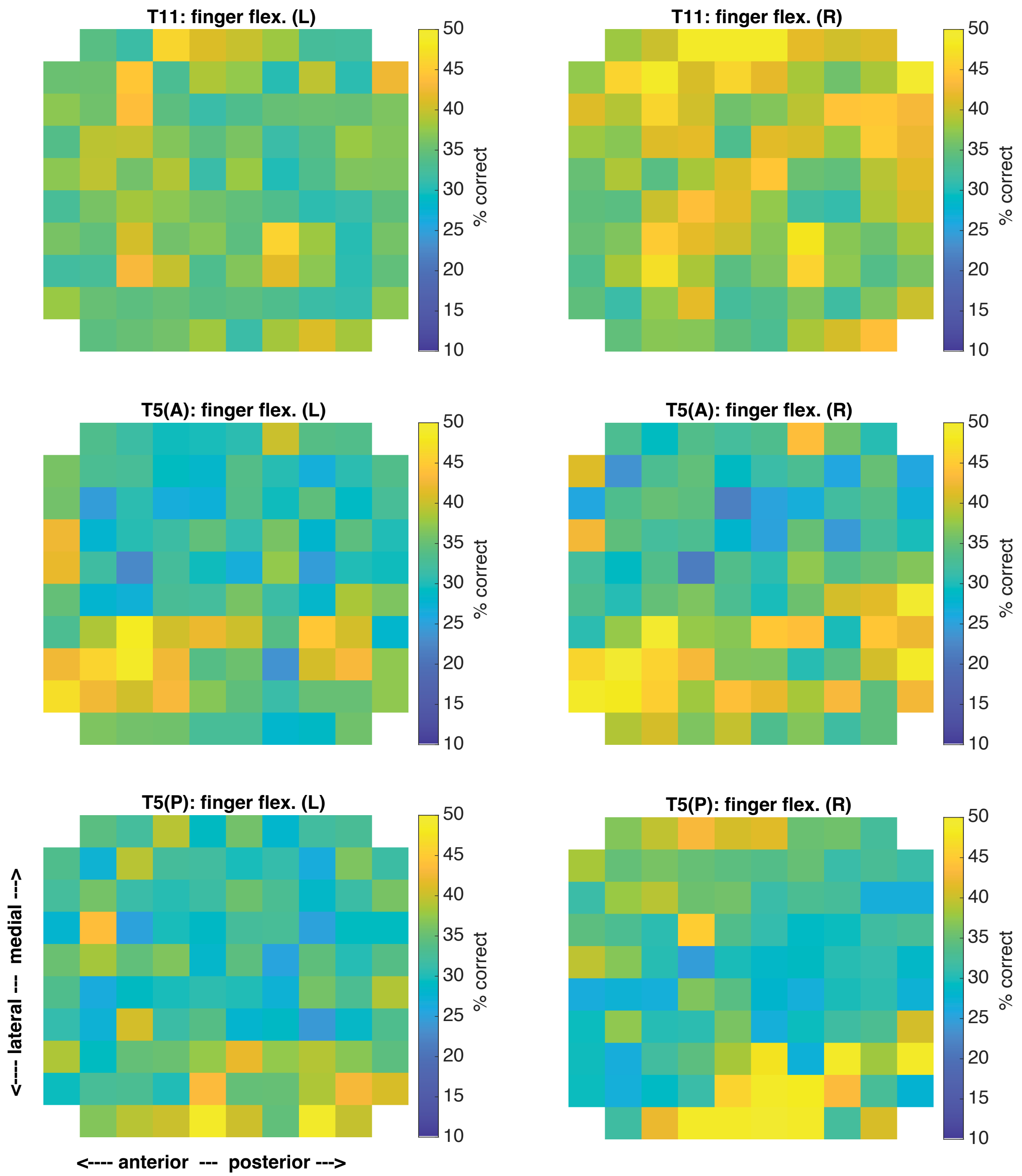

Figure S4. Finger Flexion decoding accuracy across the cortical surface. Each grid partition represents the position of a single electrode on a 10 by 10 grid (note that the corners are not connected, for a total of 96 active electrodes per array). Each square is colored according to the highest decoding accuracy for features recorded at that location (either threshold crossings or spike power) averaged across all sessions. Results for 4 way classification of individual finger flexion movements are shown separately for the left and right hands (left and right columns, respectively). Only one

array is shown for participant T11 (top row), since the other array did not display features with significant decoding. Two arrays (anterior and posterior) are shown for participant T5 (middle and bottom rows, respectively).

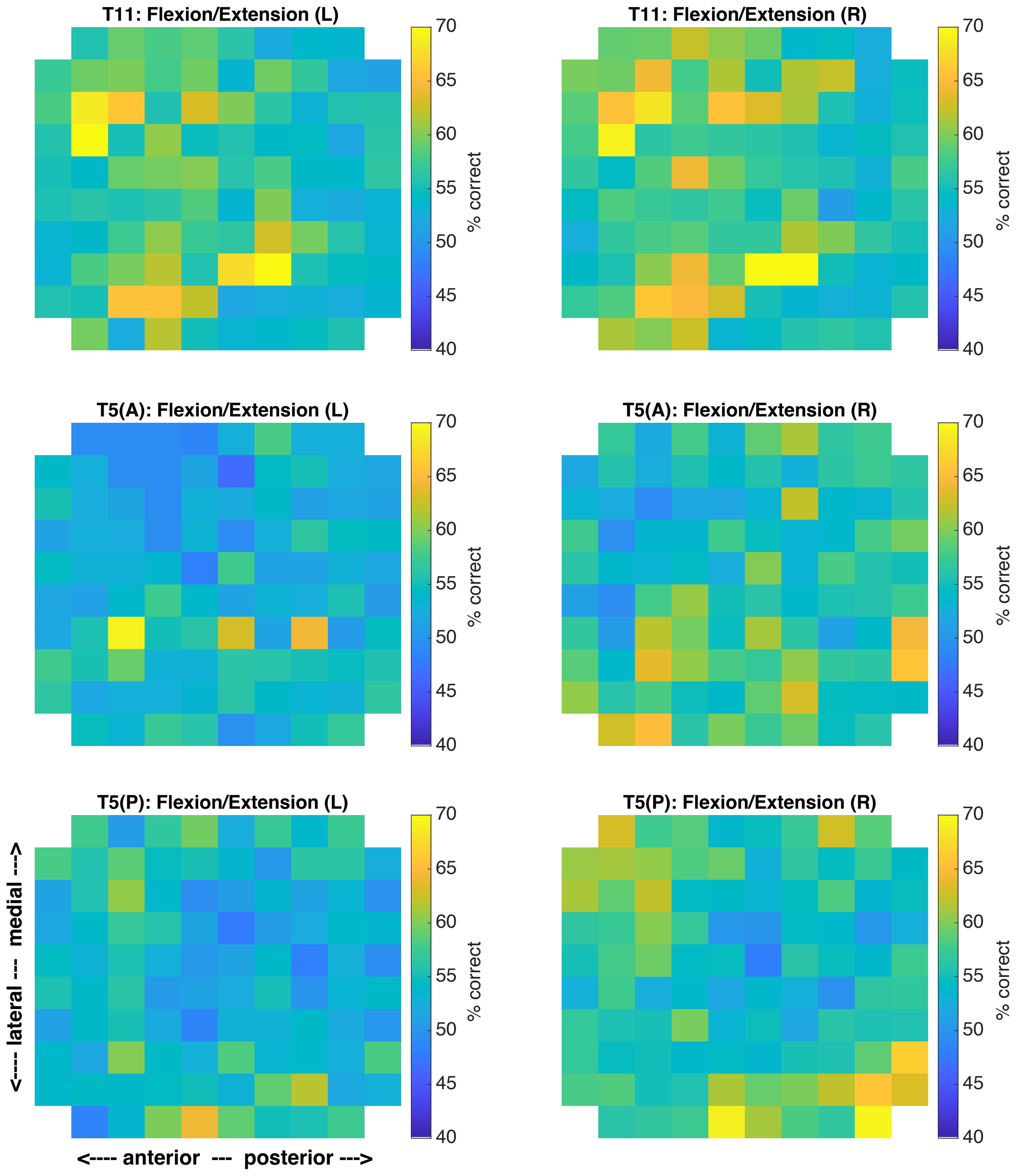

Figure S5. Flexion vs. Extension decoding accuracy across the cortical surface. Similar layout to figure S4, but showing 2-way classification of intended movements involving flexion vs. extension of the digits.

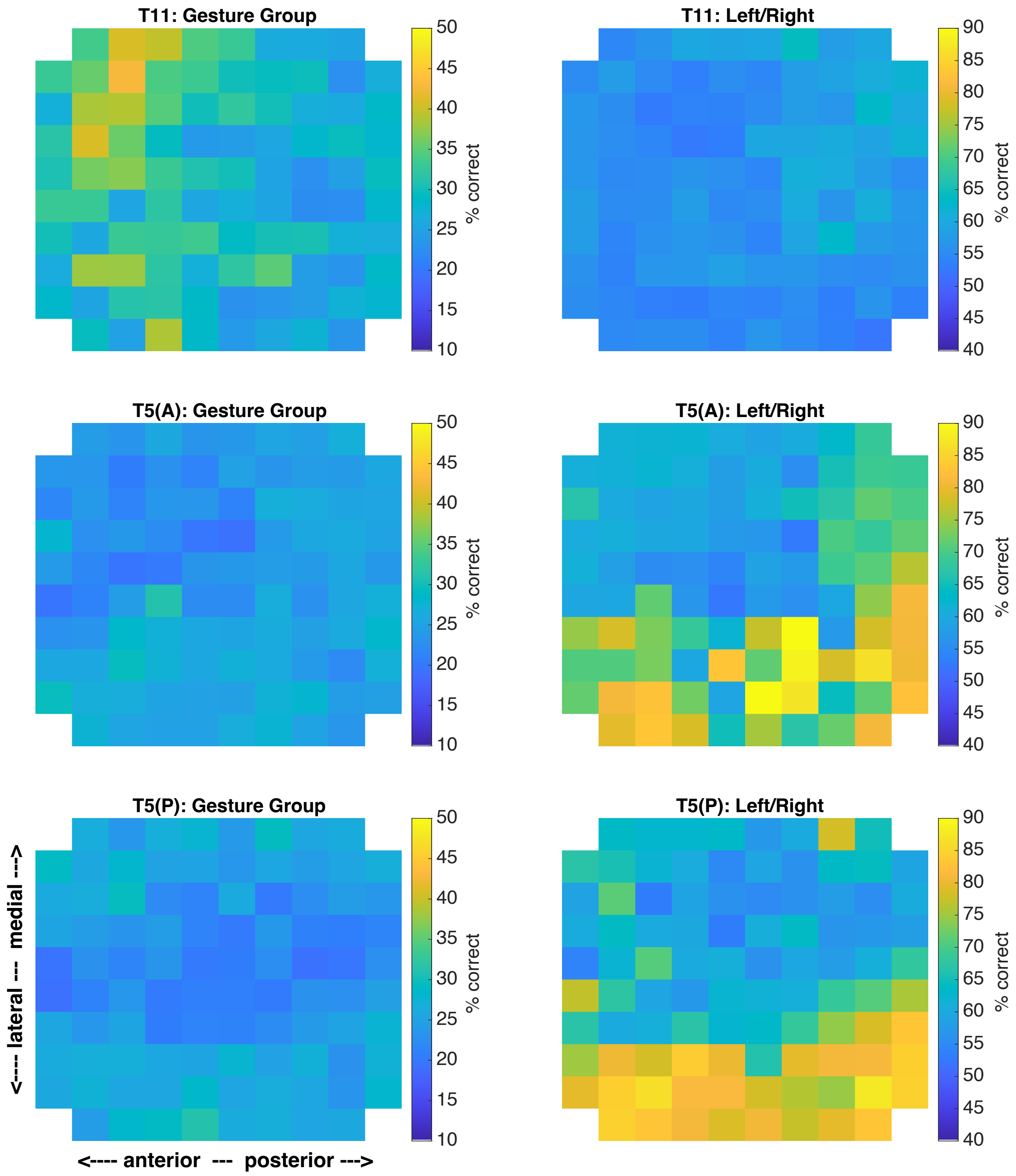

Figure S6. Gesture group / effector decoding accuracy across the cortical surface. Similar layout to figure S4, but showing 6-way classification of intended movements according to gesture class (left column) or 2-way classification of effector (i.e. left or right hand, right column).
