# Supplementary material for: Gesture encoding in human left precentral gyrus neuronal ensembles
Source: Commun Biol. 2025 Aug 30;8:1315. doi: 10.1038/s42003-025-08557-z (PMC12398507; doi:10.1038/s42003-025-08557-z)
Supplement: Supplementary file 3 — Reporting Summary [file 42003_2025_8557_MOESM3_ESM.pdf]

nature portfolio

Corresponding author(s): Carlos E. Vargas-Irwin

Last updated by author(s): Feb 4, 2025

Reporting Summary

Nature Portfolio wishes to improve the reproducibility of the work that we publish. This form provides structure for consistency and transparency in reporting. For further information on Nature Portfolio policies, see our Editorial Policies and the Editorial Policy Checklist.

Statistics

For all statistical analyses, confirm that the following items are present in the figure legend, table legend, main text, or Methods section.

n/a	Confirmed	
☐	☒	The exact sample size (*n*) for each experimental group/condition, given as a discrete number and unit of measurement
☐	☒	A statement on whether measurements were taken from distinct samples or whether the same sample was measured repeatedly
☐	☒	The statistical test(s) used AND whether they are one- or two-sided<br>*Only common tests should be described solely by name; describe more complex techniques in the Methods section.*
☐	☒	A description of all covariates tested
☐	☒	A description of any assumptions or corrections, such as tests of normality and adjustment for multiple comparisons
☐	☒	A full description of the statistical parameters including central tendency (e.g. means) or other basic estimates (e.g. regression coefficient) AND variation (e.g. standard deviation) or associated estimates of uncertainty (e.g. confidence intervals)
☐	☒	For null hypothesis testing, the test statistic (e.g. *F*, *t*, *r*) with confidence intervals, effect sizes, degrees of freedom and *P* value noted<br>*Give P values as exact values whenever suitable.*
☒	☐	For Bayesian analysis, information on the choice of priors and Markov chain Monte Carlo settings
☐	☒	For hierarchical and complex designs, identification of the appropriate level for tests and full reporting of outcomes
☐	☒	Estimates of effect sizes (e.g. Cohen's *d*, Pearson's *r*), indicating how they were calculated

Our web collection on statistics for biologists contains articles on many of the points above.

Software and code

Policy information about availability of computer code

Data collection	Data was collected using a Cerebus Neural Signal Processor and custom software (MATLAB and Simulink Real-Time MathWorks, Natick, MA) as part of the BrainGate2 pilot clinical trial (www.ClinicalTrials.gov; Identifier: NCT00912041)
Data analysis	Data analysis was carried out using MatLab version 2020 (Mathworks, Natick, MA). Code is available at https://github.com/cvargasi/GestureEncoding

For manuscripts utilizing custom algorithms or software that are central to the research but not yet described in published literature, software must be made available to editors and reviewers. We strongly encourage code deposition in a community repository (e.g. GitHub). See the Nature Portfolio guidelines for submitting code & software for further information.

Data

Policy information about availability of data

All manuscripts must include a data availability statement. This statement should provide the following information, where applicable:

- Accession codes, unique identifiers, or web links for publicly available datasets
- A description of any restrictions on data availability
- For clinical datasets or third party data, please ensure that the statement adheres to our policy

Data is available through Dryad at: https://doi.org/10.5061/dryad.cvdncjtcq

Research involving human participants, their data, or biological material

Policy information about studies with human participants or human data. See also policy information about sex, gender (identity/presentation), and sexual orientation and race, ethnicity and racism.

Reporting on sex and gender	This study included data from two participants, T5 and T11, who are both biologically male and identify as men. This information was self-reported. No sex or gender based analyses were performed given there were only two participants, and were not the main focus of this study.
Reporting on race, ethnicity, or other socially relevant groupings	No variables relating to race, ethnicity or other socially relevant groupings were reported or analyzed.
Population characteristics	The research participants (identified as T11 and T5) gave informed consent to the study and publications resulting from the research. T11 is a right-handed man, 36 years old at the time of the study, with a history of tetraplegia secondary to a cervical spinal cord injury (C4 AIS-B SCI) that occurred 11 years prior and accompanied by some cerebral injury (full history unknown); 5 years before that event, he experienced a traumatic brain injury with a subdural hemorrhage requiring surgical evacuation, with full recovery. At the time of the sessions T5 was a 68-year-old right-handed man, with a C4 AIS-C SCI that occurred approximately 9 years prior to study enrollment. Both participants had two 96-channel intracortical microelectrode arrays placed chronically into the left precentral gyrus (PCG) as part of the ongoing BrainGate2 pilot clinical trial (www.ClinicalTrials.gov; Identifier: NCT00912041). Details of the recording paradigm have been described previously (Hochberg et al. 2006, 2012; Malik et al. 2011; Simeral et al. 2021). Microelectrode array implantation targeted the “hand knob” area of the left precentral gyrus, which was identified by pre-operative magnetic resonance imaging (MRI). A description of the precise locations of the implants for T11 and T5 is provided in (Rubin et al. 2022), and (Stavisky et al. 2019), respectively. This study was performed with permissions of U.S. Food and Drug Administration (Investigational Device Exemption #G090003; CAUTION: Investigational Device. Limited by Federal Law to Investigational Use) and the Institutional Review Boards of Massachusetts General Hospital, Providence VA Medical Center, Brown University and Stanford University. All research sessions were performed at the participant’s place of residence.
Recruitment	Participant T5 and T11 were enrolled in the BrainGate2 pilot clinical trial prior to the design and execution of this study, after meeting inclusion criteria based in part on disease characteristics. Inclusion and exclusion criteria are available online (Clinica|Trials.gov, Identifier: NCT00912041). Note that this study does not report clinical trial results.
Ethics oversight	This study was performed with permissions of U.S. Food and Drug Administration (Investigational Device Exemption #G090003; CAUTION: Investigational Device. Limited by Federal Law to Investigational Use) and the Institutional Review Boards of Massachusetts General Hospital, Providence VA Medical Center, Brown University and Stanford University.

Note that full information on the approval of the study protocol must also be provided in the manuscript.

Field-specific reporting

Please select the one below that is the best fit for your research. If you are not sure, read the appropriate sections before making your selection.

☒ Life sciences ☐ Behavioural & social sciences ☐ Ecological, evolutionary & environmental sciences

For a reference copy of the document with all sections, see nature.com/documents/nr-reporting-summary-flat.pdf

Life sciences study design

All studies must disclose on these points even when the disclosure is negative.

Sample size	N/A
Data exclusions	All sessions recorded with the participants for this task were included in the analysis
Replication	Results were replicated across six sessions for participant T11 and two sessions for participant T5
Randomization	N/A
Blinding	N/A (Single Group Assignment)

Reporting for specific materials, systems and methods

We require information from authors about some types of materials, experimental systems and methods used in many studies. Here, indicate whether each material, system or method listed is relevant to your study. If you are not sure if a list item applies to your research, read the appropriate section before selecting a response.

Materials & experimental systems

n/a	Involved in the study
☒	☐ Antibodies
☒	☐ Eukaryotic cell lines
☒	☐ Palaeontology and archaeology
☒	☐ Animals and other organisms
☐	☒ Clinical data
☒	☐ Dual use research of concern
☒	☐ Plants

Methods

n/a	Involved in the study
☒	☐ ChIP-seq
☒	☐ Flow cytometry
☒	☐ MRI-based neuroimaging

Clinical data

Policy information about clinical studies

All manuscripts should comply with the ICMJE guidelines for publication of clinical research and a completed CONSORT checklist must be included with all submissions.

Clinical trial registration	www.ClinicalTrials.gov; Identifier: NCT00912041
Study protocol	https://clinicaltrials.gov/study/NCT00912041
Data collection	All research sessions were performed at the participant's place of residence.
Outcomes	N/A

Plants

Seed stocks	N/A
Novel plant genotypes	N/A
Authentication	N/A
